# Discovery of C19-9 as a novel non-RGD inhibitor of αvβ3 to overcome enzalutamide resistance in castration-resistant prostate cancer

**DOI:** 10.1038/s41392-022-01236-z

**Published:** 2023-02-10

**Authors:** Xiaocong Pang, Xiaojiao Sun, Yanlun Gu, Xu He, Kan Gong, Song Song, Jixin Zhang, Jie Xia, Zhenming Liu, Yimin Cui

**Affiliations:** 1 Peking University First Hospital, Xishiku Street, Xicheng District, 100034 Beijing, China; 2 School of Pharmaceutical Sciences, Peking University, Xueyuan Road 38, Haidian District, 100191 Beijing, China; 3 Institute of Materia Medica, Chinese Academy of Medical Sciences, Nanwei Road, Xicheng District, 100050 Beijing, China

**Keywords:** Drug screening, Drug development

**Dear Editor**,

The integrin αvβ3 receptor is a promising target for anticancer therapy.^1,2^ However, there are no effective marketed treatments targeting αvβ3. One possible limitation of Arginine-Glycine-Aspartic (RGD)-mimetic αvβ3 antagonists has been shown to cause partial agonism, which could induce major conformational changes that trigger paradoxical cell adhesion and angiogenesis.^3,4^

In this study, we identified a novel non-RGD small molecule inhibitor against the integrin αvβ3. Via virtual screening, surface plasmon resonance (SPR) assay-based affinity assay, and anti-tumor activity evaluation, C19 was identified as a hit compound with good affinity (Fig. 1a, Supplementary Figs. S1, S2, Supplementary Table S1). C19 had a good synergistic effect with enzalutamide on the inhibition of 22RV1 cell proliferation (Fig. 1b).

**Fig. 1:**
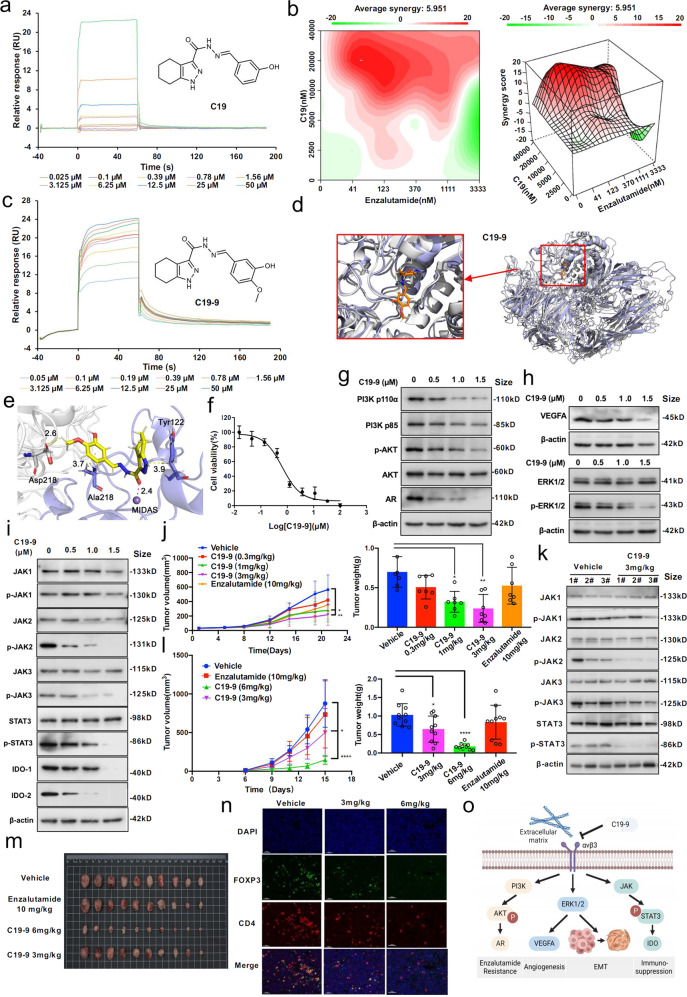
Discovery of a novel non-RGD αvβ3 inhibitor for castration resistant prostate cancer (CRPC) treatment. **a** Sensorgram of C19 interacted with αvβ3 measured by SPR method, and data was analyzed by General Electric Company(GE)‘s BIAevaluation software. The KD value was 2.68 μM. **b** The synergistic effect on inhibition of 22RV1 cell proliferation of C19 and enzalutamide. **c** The binding affinity of C19-9 with αvβ3 tested by SPR. **d** The result of superposition of molecular dynamics (MD) simulations stable structure and the crystal structure of integrin avβ3. The crystal structure is shown in light blue, and the MD stable structure is shown in grayish-white. **e** The 3D binding mode of integrin avβ3 with C19-9. The backbone and residue of the integrin avβ3 alpha chain are colored in white, and the backbone and residue of the integrin avβ3 beta chain are colored in light blue. C19-9 is colored in yellow, and the Mn^2+^ in MIDAS site is shown as a purple sphere. The surrounding residues in the binding pockets are shown as sticks. The yellow dashes represent hydrogen bond interaction, the magentas represent metal contact. **f** The Celltiter-Glo assay of evaluation of anti-tumor proliferation ability of C19-9 on prostate cancer organoid at 24 h. **g-i** The effects of C19-9 on the expression of VEGFA, p-ERK, ERK, p-PI3K, PI3K, p-AKT, AKT, AR and JAK/STAT3/IDO signaling pathway were detected by Western blot assay in 22RV1 cell line. **j** 1 × 10^6^ 22RV1 cells in suspension were subcutaneously injected into the right flank of BALB/c-nude mice. After the volume of tumor nodules reached about 75 mm^3^, tumor-bearing BALB/c-nude mice were randomly assigned to four groups (*n* = 7 per group) and treated with C19-9, enzalutamide, or vehicle as indicated. The control group was injected with DMSO. Tumor volume was measured twice per week. Tumor weight in different groups treated with C19-9 and enzalutamide after three weeks. **k** In the 22RV1-derived subcutaneous tumor growth xenograft model, tumor tissue was obtained after treatment for three weeks. The expression of JAK/STAT3 signaling pathway related proteins were tested by Western blot assay. **l** Therapeutic effects of C19-9 on castration-resistant xenografts. Murine prostate cancer TRAMP-C1 cells (1 × 10^6^) were injected subcutaneously into C57BL/6 mice. The tumor-bearing mice were castrated and randomly assigned to four groups (*n* = 10 per group). Animals were intraperitoneally injected with C19-9 (3 mg/kg and 6 mg/kg) or vehicle, and enzalutamide (10 mg/kg) by intragastric administration. The tumor volume weight was measured every 2 days. Tumor weight in different groups was measured following the termination of the experiment. **m** Image of tumors in different groups following the termination of TRAMP-C1 castration-resistant xenografts experiment. **n** Polychromatic immunofluorescence staining showing the expression of CD4 and FOXP3 in tumor tissues of C19-9 and vehicle. Bars: 50 μm. **o** The mechanism of integrin αvβ3 inhibitor C19-9 for overcoming enzalutamide resistant in CRPC. Data are expressed as mean ± SD. Statistical analyses were performed using the two-tailed Student’s *t* test to detect differences between the groups, *P-value < 0.05, **P-value < 0.01, ***P-value < 0.001

In an effort to improve the anti-tumor activity of C19, several modifications were made, which led to the discovery of C19-9 (Fig. 1c). The binding affinity of C19-9 to main integrin family members was tested by the SPR method. C19-9 had the best binding affinity to αvβ3 and the KD value was 0.102 μM (Fig. 1c). The KD value of positive control SB273005 was 0.685 μM. C19-9 also had a good affinity with αvβ5 and αvβ6, the KD values were 1.963 μM and 4.83 μM, respectively (Supplementary Fig. S3a). The KD values of C19-9 with α2bβ3 (GPIIb/IIIa), αvβ8, α5β1 and α4β7 were more than 50 μM. Microscale thermophoresis (MST) was also applied to further validate the binding affinity of C19-9. As a result, the KD value of C19-9 was 0.287 μM in the absence of Mn^2+^, different from RGD ligands, which was mostly divalent cation-dependent and C19-9 did not depend on Mn^2+^ induced conformational changes.^4^ (Supplementary Fig. S3b). Osteopontin (OPN) could interact αvβ3 and trigger outside-in integrin signaling. In the presence of Mn^2+^, OPN could interacted with αvβ3 with the KD value of 0.100 μM, but the interaction could be blocked by C19-9 (Supplementary Fig. S3b).

Molecular dynamics (MD) simulations were conducted to search for the stable complex structure of C19-9 with integrin avβ3 (Fig. 1d,e). MD simulation was performed again via alanine mutagenesis of Asp218 and Tyr122 to evaluate whether their mutation had any effect on the binding of avβ3 to C19-9. After mutation, C19-9 only formed one hydrogen bond with the oxygen atoms of residue Glu220 in the alpha chain and formed salt-bridge interaction with the manganese ion (Supplementary Fig. S4), and the ∆G total value changed from −13.469 kcal/mol to −2.991 kcal/mol, which suggested that Asp218 and Tyr122 were the key amino acids for the C19-9 interaction affinity with avβ3 (Supplementary Table S2). Interacting with Tyr122 in the beta chain plays an important role in preventing the conformational change in αvβ3.^4^ Our homology modeling assays suggested that C19-9 could also interact with Phe122 in mouse beta 3 chain (corresponding to Tyr122 in human) and MIDAS site (Supplementary Fig. S4d–f). The benzene ring in C19-9 forms Pi-H interaction with the carbon atoms of residue Tyr122 in the beta chain (Fig. 1e), which could block the movement toward the metal ion-dependent adhesion site (MIDAS), a key element in triggering the conformational change, suggesting that C19-9 could be a pure antagonist of αvβ3.

The Celltiter-Glo assay showed that the IC_50_ of C19-9 on 22RV1 at 24 h was 0.467 μM. We also tested the inhibitory effect of C19-9 on PCa organoids and the results of the assay showed that C19-9 significantly inhibited organoid proliferation in a dose-dependent manner with IC_50_ of 0.652 μM (Fig. 1f). C19-9 significantly reduced the adhesion and migration of PC-3 cells in a dose-dependent manner (Supplementary Fig. S5a, b). C19-9 also could inhibit the HUVECs tube formation with a concentration ranging from 0.3 μM to 1 μM in the tumor organoid-induced angiogenesis assay (Supplementary Fig. S5c).

To explore the mechanism of C19-9 inhibition of castration resistant prostate cancer (CRPC) development, we analyzed the changes of differential genes after 22RV1 cells disposed of C19-9. The GO and KEGG enrichment pathway had a high correlation with the signals related to integrin αvβ3, such as cell adhesion, angiogenesis, extracellular matrix organization, PI3K/Akt pathway, tryptophan metabolism, etc. (Supplementary Fig. S6). C19-9 could inhibit PI3K/Akt signaling pathway and decrease the expression of AR (Fig. 1g). C19-9 could inhibit epithelial lineage differentiation by enhancing the expression of E-cadherin and decreasing N-cadherin, and Vimentin (Supplementary Fig. S7a). In addition, C19-9 could inhibit 22RV1 secreting VEGFA and phosphor-ERK (p-ERK) in a concentration-dependent manner (Fig. 1h), which was further validated in 22RV1 derived castration-resistant xenografts (Supplementary Fig. S8).

Differential expression genes for tryptophan metabolism are mainly referred to IDO1 and IDO2, significantly downregulated in 22RV1 cells treated with C19-9. Upregulation of IDO expression has been detected in murine PCa tumors and in advanced PCa patients,^5^ which also validated in our study, and C19-9 could inhibit IDO expression significantly in PCa organoid KOPCa-032 (Supplementary Fig. S7b, c). JAK/STAT3 signaling pathway is known to activate the transcription of IDO. From Fig. 1i–k, we found C19-9 negatively regulated the JAK/STAT3 signal transduction pathway and decreased IDO1 and IDO2 expression in vivo and in vitro, and especially decreased the expression of pJAK2, pSTAT3 in a concentration- and dose-dependent way.

C19-9 demonstrated robust anti-tumor activity in vivo and displayed good safety and stability. The efficacy of C19-9 was evaluated in vivo by treating 22RV1 xenografts in male BALB/c nude mice for 21 days, with comparison with the well-known AR-antagonist enzalutamide. Intraperitoneal administration of 1 and 3 mg/kg per day, C19-9 significantly inhibited the increase of tumor volume in 22RV1 xenografts (Fig. 1j, Supplementary Fig. S8a). In contrast, the CRPC 22RV1 xenografts were resistant to enzalutamide administration. In addition, C19-9 did not significantly affect the body weight of mice in acute and long-term toxicity experiments, and did not show apparent toxicity according to the pathological review of sections of the heart, liver, lung, and kidney (Supplementary Fig. S9).

Administration of C19-9 had excellent efficiency in blocking the enzalutamide-resistant growth of murine CRPC xenografts. As shown in Fig. 1l, m, C19-9 with 6 mg/kg administration decreased the tumor weight by 85% (95% confidence interval.64–100%), whereas enzalutamide treatment reduced the tumor weight by only 19% (95% confidence interval.-17%–56%). Immunohistochemical staining of Ki67, CD31, Vimentin, N-cadherin, and E-cadherin in the xenograft tumors (Supplementary Fig. S10), which further verified that C19-9 could inhibit tumor proliferation, angiogenesis and EMT. In addition, C19-9 could up-regulate the expression of TNFα, IFN-γ, and IL-2 in a dose-dependent way, and also increase IL-12 expression, while the immunosuppressive cytokine IL-1β and CXCL1 expression were significantly down-regulated at C19-9 administration with the dose of 3 mg/kg and 6 mg/kg (Supplementary Fig. S11). Here we also showed that CD4 + FOXP3 + Treg cell was remarkably decreased in C19-9 3 mg/kg and 6 mg/kg groups (Fig. 1n).

The LogD7.4 of C19-9 was 1.66, which indicated C19-9 had satisfying solubility and cell permeability. In addition, in vitro metabolic stability test also suggested that C19-9 had good plasma stability (t1/2 > 8 h) and high liver microsomes stability (only 5% C19-9 degradation after incubation for 4 h) in different species.

In conclusion, we identified C19-9, a novel non-RGD inhibitor of αvβ3 with excellent drug-like characteristics that could overcome enzalutamide-resistance in CRPC via inhibiting EMT and angiogenesis, blocking PI3K/Akt/AR and EKR signaling pathways, and improving the immunosuppressive microenvironment by JAK/STAT3/IDO signaling pathway (Fig. 1o). Taken together, C19-9 can be regarded as a novel potential drug candidate for CRPC treatment.

## Supplementary information

Supplementary_Materials-clean

## Data Availability

The data that support the findings of this study are available from the lead corresponding author upon reasonable request.
