# Supplementary material for: Discovery of C19-9 as a novel non-RGD inhibitor of αvβ3 to overcome enzalutamide resistance in castration-resistant prostate cancer
Source: Signal Transduct Target Ther. 2023 Feb 10;8:60. doi: 10.1038/s41392-022-01236-z (PMC9911763; doi:10.1038/s41392-022-01236-z)
Supplement: Supplementary file 1 — Supplementary_Materials-clean [file 41392_2022_1236_MOESM1_ESM.docx]

Supplementary Materials for

Discovery of C19-9 as a novel non-RGD inhibitor of αvβ3 to overcome enzalutamide resistance in castration-resistant prostate cancer

Xiaocong Pang^1,2#^, Xiaojiao Sun^3#^, Yanlun Gu^1,2#^, Xu He ^1,2^, Kan Gong^4^, Song Song^3^, Jixin Zhang^5^, Jie Xia^6^, Zhenming Liu ^3*^, Yimin Cui ^1,2^*

Correspondence to: Yimin Cui (e-mail: cui.pharm@pkufh.com), Zhenming Liu (e-mail: zmliu@bjmu.edu.cn).

**This PDF file includes:**

Materials and Methods

Figures. S1 to S12

Tables S1 to S2

Materials and Methods

1. Synthesis of C19-9

General methods. All reagents and solvents were purchased from commercial sources and used as obtained. ^1^H NMR and ^13^C NMR spectra were recorded with a Bruker 400 NMR spectrometer and referenced to deuterium dimethyl sulfoxide (DMSO-*d_6_*). Chemical shifts were expressed in ppm. In the NMR tabulation, s indicates singlet; d, doublet; t, triplet; q, quartet; m, multiplet; and br, broad peak. Mass spectra was measured with SCIEX Triple TOF 6600+ system using an ESI source operating in positive ion mode, coupled to a SCIEX Exion LC AD system. The purity of all compounds were above 95% purity as determined by a SHIMADZU LC-2010A HT HPLC with UV detection at 254 nm. Melting points were determined on a M-560 melting point apparatus from BÜCHI Labortechnik AG.


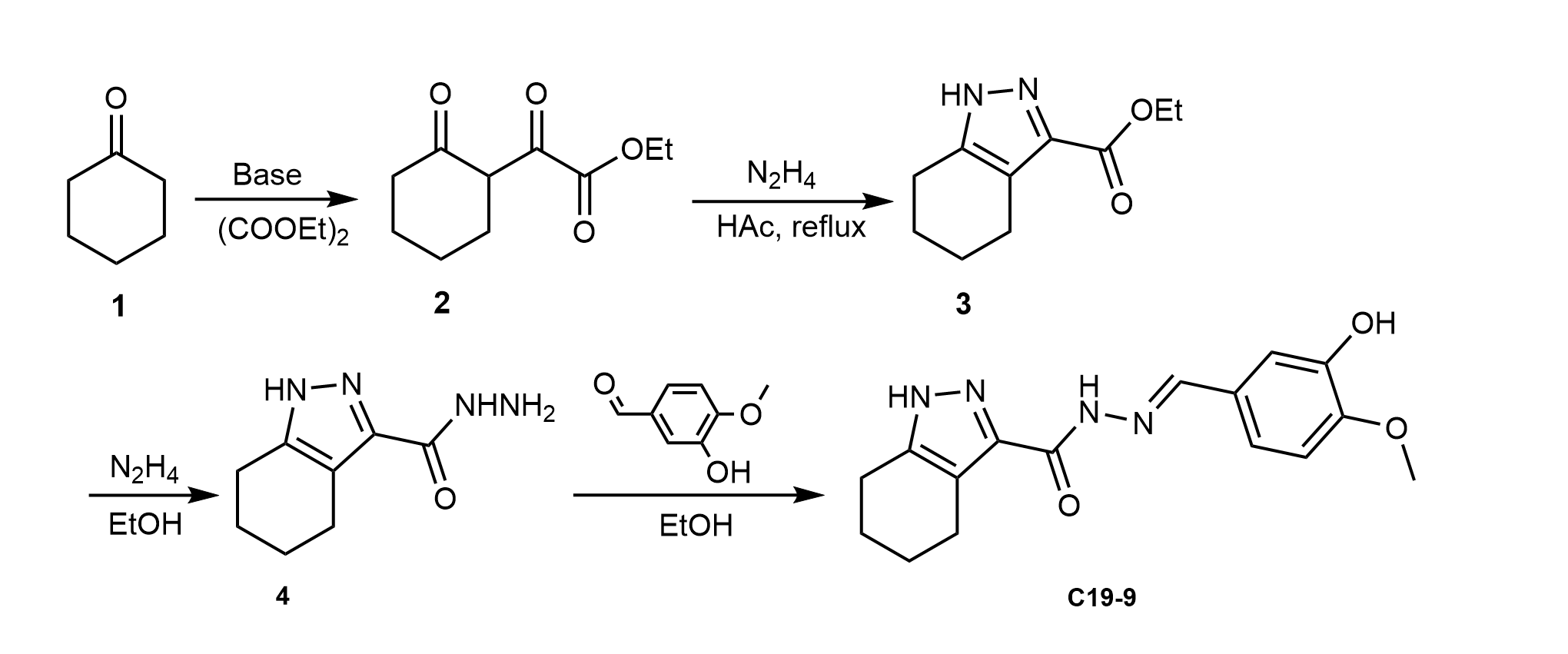


A solution of sodium ethoxide was prepared by adding sodium (1.5 g, 65 mmol) to anhydrous ethyl alcohol (20 mL) at 0oC. The mixture of cyclohexanone (4.41g, 44 mmol) and diethyl oxalate (7.3 g, 50 mmol) was then slowly added and the solution was stirred for 12h at room temperature. Following decomposition of the reaction mix with 2N sulfuric acid solution, the mixture was extracted with ethyl acetate and the organic solvents were dried and concentrated. The crude product was further purified by column chromatography with n-hexane: ethyl acetate (12:1) to afford **2** (5.87 g, **yield=67%**) as a yellow oil. Next, hydrazine (448 mg, 14 mmol) was slowly added to a cooled suspension of compound **2** (2.38 g, 12 mmol) in acetic acid (5 mL). The mixture was heated to reflux for 1 h, poured into ice-H2O, neutralized with NaHCO_3_, and extracted with ethyl acetate. The combined organic layers were dried (Na_2_SO_4_), filtered, and concentrated. The residue was purified by column chromatography with n-hexane: ethyl acetate (3:1) to yield **3** (2 g, **yield=86%**) as a white solid. Ester **3** (970 mg, 5 mmol) and hydrazine (1.34 g, 35 mmol) in ethanol (10 mL) was next heated to reflux for 1day. At the end of this period, ethanol was evaporated, and the precipitate was collected by filtration, washed with ethyl acetate and water to give hydrazide **4** as a white solid (580 mg, **yield=64%**). Next**,** hydrazide **4** (72 mg, 0.4 mmol) and substituted aldehydes (0.44 mmol) in ethanol (3 mL) were refluxed to obtain C19-9 as a precipitate. The precipitate was then collected by filtration and washed with cold ethanol (**yield=81%**) as a light yellow solid.Purity:100.0%: ^1^H NMR (400 MHz, DMSO-*d*_6_) δ 12.88 (s, 1H), 11.30 (s, 1H), 9.28 (s, 1H), 8.32 (s, 1H), 7.21 (s, 1H), 7.02 – 6.88 (m, 2H), 3.79 (s, 3H), 2.70 – 2.56 (m, 4H), 1.80 – 1.61 (m, 4H). ^13^C NMR (101 MHz, DMSO-*d*_6_) δ 159.02, 149.49, 146.85, 146.82, 141.30, 139.97, 127.57, 119.91, 116.58, 112.26, 111.86, 55.56, 22.64, 21.99, 21.04, 20.59; LC-MS (ESI, m/z): 315.1463[M+H]^+^; Molecular weight: 315.3450; Melting point: 261.0℃

2. Machine learning and molecular docking simulations for *in silico* virtual design αvβ3 inhibitor

The Specs database (http://www.specs.net) was used as the screening dataset. The “Prepare Ligands” protocol in Discovery Studio 2016 was used to prepare the Specs chemical library and filtered by Lipinski’s “rule of 5”. Two machine learning (ML) algorithms were explored in this study: SVM and Random forest (RF). The workflow of ML models establishment was shown in supplement Fig.S1. A total of 1,337 unique compounds annotated with IC_50_ values for human αvβ3 were compiled from the Pubchem database, of which ten percent were held back as an external dataset, and the remaining compounds were randomly divided into the training set and test set at a ratio of 4:1. And 182 molecules annotated with IC_50_ values were downloaded from Chembl for database for the external validation set. Those compounds with the IC_50_ values below 1 μM were defined as active, while all the others were regarded as inactive. Morgan2 fingerprints were calculated with RDKit (version 2019.9.3) and used for modeling. For evaluating the performances of the models, AC, SE, SP, MCC, ROC AUC were measured based on 10-fold cross-validation, test set and external test set validation. Molecule docking was conducted by the FRED program (OpenEye Scientific Software, Inc., Santa Fe, NM, USA). The X-ray structure of integrin αvβ3 (PDB ID: 1L5G and 6MK0) were downloaded from the protein data bank (PDB) database. The binding poses of each compound were scored by Chemgauss4, the inherent scoring function of FRED. The scores were ranked according to the FRED. The top-ranking compounds based on Chemgauss4 scores with good binding mode were selected for further study. These compounds, including C19, C23, and C25 were purchased from Specs Compound Handling B.V.(Bleiswijkseweg 55, 2712 PB Zoetermeer, The Netherlands)

3. Surface Plasmon Resonance (SPR) and MicroScale Thermophoresis (MST) Assay-based integrin αvβ3 inhibitor affinity assay

To inspect the potential hits with binding affinities to integrin αvβ3, αvβ5, α2bβ3, αvβ6, αvβ8, α5β1, and α4β7, SPR technology-based Biacore 8K was applied according to the manufacturer’s instruction. Briefly, integrin proteins were conjugated to the CM5 chip by the amine-coupling method. The bound ligand was diluted in HBS-P buffer (0.01M HEPES pH7.4，0.15M NaCl，0.05% v/v Surfactant P20) and injected into the flow cells at a flow rate of 10 μL/min. The running buffers were used as a negative control and the small molecule, SB273005 with a high binding affinity to integrin αvβ3 was used as a positive control. The sensor surfaces were regenerated with a short pulse of 10 mM glycine, pH 2.2. All SPR measurements were performed at 25 °C. Data were analyzed by the Biacore 8K Control Software for ligand-integrin αvβ3 binding affinity. According to the 1:1 Langmuir binding fit model provided by General Electric Company(GE)'s BIAevaluation software to fit the data obtained from the SPR experiment, de dissociation rate constant (Kd), the association rate constant (Ka), and equilibrium dissociation constant (KD) were calculated. KD is the quotient of kd and ka, which is the concentration of the analyte where 50% of the ligand is occupied by the analyte in a 1 to 1 interaction.

MST experiments were performed on a Monolith NT.115pico (NanoTemper Technologies GmbH, Munich, Germany). Integrin αvβ3 protein (200 nM) was labeled with Monolith NT^TM^ Protein Labeling Kit Red-NHS in HBS-P buffer (0.01M HEPES pH7.4，0.15M NaCl，0.05% v/v Surfactant P20). The human OPN was diluted in HBS-P buffer with αvβ3 MnCl_2_ and the C19-9 was diluted in HBS-P buffer with or without MnCl_2_. To study the effect of C19-9 on the binding ability of OPN, αvβ3 incubated with C19-9 for 2h was pretreated before MST test.10 µL diluted ligand derivates at various concentrations were mixed with 10 μL labeled protein, and then were loaded to standard capillaries. Automated MST measurement was carried out with Medium MST power. KD value was calculated based on a dose-response curve using MO. AffinityAnalysis software (NanoTemper Technologies GmbH).

4. Cell culture

Prostate cancer cell lines PC-3, DU-145, 22RV1, and other tumor cell lines U87, WM115, and OVCAR-4 were purchased from the Cell bank of the Chinese Academy of Science (Shanghai, China). 22RV1 and OVCAR-4 cell lines were cultured in RPMI-1640 medium. PC-3 and DU-145 cell line was cultured in F-12K Medium and Eagle's Minimum Essential Medium, respectively. U87, WM115 cells were maintained in Dulbecco’s modified Eagle’s medium (DMEM). Media were supplemented with 10% FBS and 1% penicillin/streptomycin. Cell culture was at 37°C with a 5% CO_2_-humidified atmosphere.

5. Cell viability assay

Cell viability test was performed by Celltiter-Glo assay (Promega P/N G7570). In brief, tumor cells were cultured into 96-well plates (1.2 x 10^4^ cells/ml) and incubated overnight. The cells were treated with media or compounds for 72 h. Chemiluminescence method was used to measure cell ATP level to evaluate cell viability. The specific operation is in accordance with the instructions. After the incubation is completed, add 50μL CTG solution, and after mixing, transfer the lysis mixture to the microplate reader. After 5-10 minutes, collect the chemiluminescence data in the microplate reader. GraphPad Prism 7 software was used to calculate the IC_50_ value based on the fitted pharmacodynamic curve of chemiluminescence data.

6. Cell adhesion assay

To investigate the potential effect of C19-9 on the ability of cells to adhere, the PC-3 cell lines were treated with C19-9 at three concentrations in triplicate. Firstly, prepare 40 μg/ml Collagen I solution in PBS, store at 4 °C; prepare 0.1% BSA solution in DMEM, and coat the 96-well plate with the 10% FN at 4 °C. After 12 h of coating, remove the FN and air-dry the plate at room temperature in the tissue-culture hood. Use 0.05% Trypsin to detach the cells and then observe them under a microscope to confirm the complete dissociation of the cells. After washing cells, add 90 μl cell suspension to each of the FN-coated wells and add 10 μl C19-9 to responded wells. Incubate the plate at 37 °C for 24-48h to allow the cells to adhere to the surface. Adherent cells were fixed with 50 μL of 96% ethanol for 10 min, stained with 50 μL of 0.1% crystal violet, rinsed extensively with water, and dried at room temperature. Stained cells were solubilized with 50 μL of 0.2% Triton X-100 and quantified by measuring the absorbance at 570 nm.

7. Cell migration assay

Cell migration was evaluated by in vitro wound-healing assay. Cells were seeded in a 6-well plate. The wound was generated by scratching the Confluent monolayers with a 200-µl pipette tip. The monolayer was washed with PBS to remove the detached cells, and was treated with different concentrations of C19 and its derivates, and incubated for 24h and 48 h at 37˚C. The cell movement into the wounded area was quantified via measuring the width of the cell-free zone at six distinct positions. All micrographs were captured at the same time and magnification.

8. Establishment of organoid system

The organoid system was built from primary PCa tissues. In brief, biopsy tissues were obtained from patients with advanced prostate cancer after ethical approval. The tissues were washed with cold PBS containing antibiotics and chopped into small pieces with surgical scissors. Tissues were further washed with 10 mL Advanced DMEM/F12 and digested in 10 mL Advanced DMEM/F12 containing 2% FCS and 2 mg/ml collagenase (Sigma, C9407) on an orbital shaker at 37°C for 0.5–1 h. The pellet was resuspended in 10 ml Advanced DMEM/F12 containing 2% FCS and centrifuged again at 400 rcf. Dissociated cells were collected in Advanced DMEM/F12 (Thermo Fisher Scientific, Waltham, MA, USA), suspended in growth factor reduced (GFR) matrigel (Corning Inc., Corning, NY, USA), and seeded. The matrigel was then solidified and overlaid with 500 μl of the complete human organoid medium, which was subsequently refreshed every two days. PDOs were cultured in Advanced DMEM/F12, supplemented with 1x B27 additive and 1x N2 additive (Thermo Fisher Scientific, Waltham, MA, USA), 0.01% bovine serum albumine, 2 mM L-glutamine, 100 units/ml penicillin-streptomycin, and containing the following additives: EGF, noggin, R-spondin 1, gastrin, FGF-10, FGFF-basic, Wnt-3A, prostaglandin E2, Y-27632, nicotinamide, A83-01, SB202190, HGF (Pepro-Tech, London, UK). Passaging of PDOs was performed using TrypLe. PDOs were biobanked in FBS (Thermo Fisher Scientific, Waltham, MA, USA), containing 10% DMSO (Sigma- Aldrich, St. Louis, MI, USA). The organoids were cultured in a cell culture incubator at 37°C and 5% CO2. The culture was replenished with fresh media every 3–4 days during organoid growth.

9. In vitro tumor organoid-induced angiogenesis assay

Tumor organoid cells (2.5 x 10^4^ cells/well) and HUVECs (2.5 x 10^4^cells/well) were co-cultured in 24-well plate at 37°C in incubator shakers and placed for 24 hours to form a suspension of Spheroids. The 24-well plate was coated with 40 mL 10% Matrigel matrix for 45 min at 37℃. 40uL Spheroids suspension were seeded on the Matrigel bed and treated with C19-9 at the indicated concentration containing 200 ng/ml VEGF or medium after 24h. After 2 days, 5 days, and 7 days, tube formations were recorded with an inverted microscope and the tubular structures were counted manually.

10. Western blotting and cytokine measurement

Total protein was extracted from 22RV1 cell lines or the xenograft tumors, and examined by Western blot following our previously published protocols. In brief, cells or tumor tissue were lysed on ice with RIPA buffer and centrifuged at 4˚C. Equivalent amounts of protein (50 μg) were separated with 10% SDS-PAGE and transferred to PVDF membranes (Millipore, Bedford, MA, USA). Membranes were blocked with 5% milk in TBST and then incubated with primary antibodies overnight at 4°C: anti-vimentin (Cell Signal Tech, Danvers, USA), anti E-cadherin (Cell Signal Tech, Danvers, USA), anti-N-cadherin (Cell Signal Tech, Danvers, USA), anti-pERK1/2 (Cell Signal Tech, Danvers, USA), anti-ERK1/2 (Cell Signal Tech, Danvers, USA), anti-IDO1 (Abcam, Cambridge, UK), anti-IDO2 (Abcam, Cambridge, UK), anti-STAT3 (Abcam, Cambridge, UK), anti-phopho-STAT3 (Abcam, Cambridge, UK), VEGFA (Abcam, Cambridge, UK), anti-PI3K p110α (Cell Signal Tech, Danvers, USA), anti-PI3K p85 (Cell Signal Tech, Danvers, USA), anti-AKT (Cell Signal Tech, Danvers, USA), phopho-AKT (Ser 473) (Cell Signal Tech, Danvers, USA), anti-AR (Cell Signal Tech, Danvers, USA) and anti-β-actin (Proteintech, Chicago, USA). JAK1, JAK2, and JAK3 monoclonal antibody (Cell Signal Tech, Danvers, USA). Phospho-JAK1 and Phospho-JAK3 polyclonal antibody were purchased from ImmunoWay Biotechnology. anti-JAK2 Phospho (Tyr1007/Tyr 1008) monoclonal antibody was purchased from Arigobio. After washing with PBS, the membranes were incubated with HRP conjugated goat-anti-mouse or goat-anti-rabbit secondary antibodies. The immunoreactive bands were visualized with BeyoECL Plus (Beyotime, P0018S). The various cytokine levels were determined by Luminex Assays (R&D Systems).

11. Immunohistochemical staining and polychromatic immunofluorescence staining

The details of immunohistochemical staining procedures are described in our previous study. Briefly, sections were blocked with 3% normal goat serum and incubated with primary antibodies overnight at 4°C. The primary antibodies used for immunohistochemical staining were Ki67, CD31, Vimentin, N-cadherin, E-cadherin, IDO1, and IDO2 (Abcam, Cambridge, UK). Sections were visualized with staining suitable secondary antibodies followed by avidin-biotin-peroxidase complex. Polychromatic immunofluorescence staining was conducted by utilizing the four color multiple fluorescent immunohistochemical staining kit (abs50012, Absin, Shanghai, China) referring to the Tyramide signal amplification (TSA) technique according to the manufacturer’s manual.

12. *In vivo* subcutaneous tumor growth xenograft model

BALB/c-nude mice (6–8-weekold, male) were obtained from Beijing Huafukang Biotechnology Co., Ltd. and housed in the animal care facility. The animal use protocol was approved by the Institutional Animal Care and Use Committee of Peking University Health Science Center. The 22RV1 xenograft tumor model was developed by subcutaneously injecting 1 x 10^6^ 22RV1 cells in suspension. When tumor nodules were developed to a volume of about 75mm^3^, tumor-bearing BALB/c-nude mice were randomly assigned to four groups and treated with C19-9, enzalutamide, or vehicle . The tumor volume and mouse body weight were measured twice a week. Tumor volume was computed by the following formula: Volume = (*d*1 × *d*2 × *d*3) × 0.5236, and *dn* means the three orthogonal diameter measurements.

13. *In vivo* murine castration-resistant prostate cancer model

Murine prostate cancer TRAMP-C1 cells (1x10^6^) were injected subcutaneously into C57BL/6 mice (aged 6–8 weeks). The tumor-bearing mice were castrated and randomly assigned to four groups. Animals were intraperitoneally injected with C19-9 (3mg/kg and 6mg/kg) or vehicle, and enzalutamide (10 mg/kg) by intragastric administration. The tumor volume and mouse body weight were measured every 2 days. Tumor weight was obtained following the termination of the experiment. Tumor volume measure was the same as BALB/c-nude mice tumor growth xenograft model.

14. Toxicity studies in mice

Toxicity studies of C19-9 after a single dose of 10mg/kg and 30mg/kg and with the dose of 3mg/kg, 10mg/kg, and 30mg/kg intraperitoneal administered continuously were evaluated at 2 weeks. Body weight and clinical pathology were monitored throughout the study. A necropsy was conducted on the day after the last administration. To measure the pathological score, ten non-overlapping fields of view were selected randomly under the microscope and were scored using a semiquantitative pathological assessment method[1-3]. All sections were scored blindly by two experienced pathologists under a light microscope according to the criteria as follows. Kidney: (1) normal kidney morphology or denotes rare necrosis (necrosis of <5% of renal tubules), (2) denotes mild necrosis (necrosis of 5-25% of renal tubules), (3) denotes moderate necrosis (necrosis of 25-75% of renal tubules), and (4) denotes severe necrosis (necrosis of >75% of renal tubules). Lung: (1) normal tissue or minimal inflammatory change; (2) no obvious damage to the lung architecture; (3) thickening of the alveolar septae; (4) formation of nodules or areas of pneumonitis that distorted the normal architecture; and (5) total obliteration of the field. Liver: The morphological liver integrity was graded on a scale of 1 (excellent) to 5 (poor). Grading is described as: (1) normal rectangular structure, (2) rounded hepatocytes with an increase of the sinusoidal spaces, (3) vacuolization, (4) nuclear picnosis, and (5) necrosis. Heart: The morphological heart integrity was graded on a scale of 1 (excellent) to 5 (poor), based on the degree of myocardial fiber morphological disorder and the degree of myofibrillar interstitial edema.

15. Molecular dynamics simulation

Molecular docking was conducted in MOE v2020.0901. The X-ray structure of integrin avβ3 was downloaded from RCSB Protein Data Bank (PDB ID: 6NAJ). The position of the original ligand in the crystal structure of integrin avβ3 was chosen as the binding site for molecular docking. Prior to docking, the force field of AMBER10:EHT and the implicit solvation model of Reaction Field (R-field) were selected. The docking workflow followed the “induced fit” protocol, in which the side chains of the receptor pocket were allowed to move according to ligand conformations, with a constraint on their positions. The final best ranked pose was selected and for further all-atom, explicit water molecular dynamics (MD) simulation. The complex of C19-9 with integrin avβ3 after docking was optimized by MD simulation. MMFF94x Force Field parameters were used for C19-9. Hydrogen atoms of C19-9 were optimized by Gaussian16 package at the level of HF/6-31g*. Then the partial atomic charges were calculated by the restrained electrostatic potential (RESP)[^4^](#_ENREF_4) charge from the calculation with Gaussian16 package at HF/6-31g* level. Then the complex was neutralized by adding sodium/chlorine counter ions and solvated in a cuboid box of TIP3P water molecules with solvent layers 8 Å between the box edges and solute surface.

All MD simulations were performed using AMBER16. The AMBER GAFF and FF14SB force fields were applied and the SHAKE algorithm was used to restrict all covalent bonds involving hydrogen atoms with a time step of 2 fs. The Particle mesh Ewald (PME) method was employed to treat long-range electrostatic interactions. For each solvated system, two steps of minimization were performed before the heating step. The first 4000 cycles of minimization were performed with all heavy atoms restrained with 50 kcal/(mol·Å2), whereas solvent molecules and hydrogen atoms were free to move. Then, non-restrained minimization was carried out involving 2,000 cycles of steepest descent minimization and 2,000 cycles of conjugated gradient minimization. Afterwards, the whole system was first heated from 0 K to 300 K in 100 ps using Langevin dynamics at a constant volume and, then, equilibrated for 150 ps at a constant pressure of 1 atm. Periodic boundary dynamics simulations were carried out for the whole system with an NPT (constant composition, pressure, and temperature) ensemble at a constant pressure of 1 atm and 300 K in the production step. In the production phase, a 100 ns simulation was carried out. The binding free energy of the complex was calculated using the MM-PBSA method.

We performed alanine mutagenesis of D218A on the av domain and Y122A on the β3 domain through MOE to test whether Y122 mutation had any effect on the binding of the avβ3 to C19-9. The complex of C19-9 with mutated integrin avβ3 after docking was optimized by MD simulation, which was similar to the above description.

Homology modeling of murine avβ3: Models are computed by the SWISS-MODEL server homology modeling pipeline, which relies on ProMod3, a comparative modeling engine based on OpenStructure. ProMod3 extracts initial structural information from the template structure. Insertions and deletions, as defined by the sequence alignment, are resolved by first searching for viable candidates in a structural database. Final candidates are then selected using statistical potentials of mean force scoring methods. If no candidates can be found, a conformational space search is performed using Monte Carlo techniques. Non-conserved side chains are modeled using a backbone-dependent rotamer library. The optimal configuration of rotamers is estimated using the graph-based TreePack algorithm by minimizing the SCWRL4 energy function. As a final step, small structural distortions, unfavorable interactions, or clashes introduced during the modeling process are resolved by energy minimization. ProMod3 uses the OpenMM library to perform the computations and the CHARMM27 force field for parameterization. Homology model of the mouse integrin AVB3 is built, using the crystal structure of protein superoxide dismutase [Protein Data Bank (PDB) code: 1L5G] as a template.

16. LogD evaluation

The determination of the distribution coefficient (LogD) was performed using a modified version of the shake-flask method. Before the experiment, octanol and phosphate buffer (PBS pH 7.4) were mixed together for 24 h to allow saturation of each solution. The mixture was allowed to rest, and the phases were separated and used as solvents in the coefficient measurement. The experiment was performed at room temperature using triplicates for each measurement. C19-9 (0.1 mg) was placed in a vial to which saturated PBS (1 mL) and octanol (0.5 mL) were added. The vial was then shaken mechanically for 10 min. The mixture was allowed to rest for 30 min or until phase separation was completed. The area under the curve (AUC) of the corresponding peak was integrated for each phase injected.

17. *In vitro* plasma and liver microsome stability

Liquid chromatography-tandem mass spectrometry (LC-MS/MS) was applied to the detection of the drugs in plasma and liver microsomes. The high-performance liquid chromatography system (Shimadzu LC-20AD) consisted of a degasser, a binary pump, and an autosampler. Electrospray ionization mass spectrometry was performed on an API Qtrap 5500 mass spectrometer (Applied Biosystems Inc., USA). Sildenafil was used as the internal standard (IS). The m/z for C19-9 was 457.3 → 58.1, while that for Sildenafil was 316.1→ 149.1. Calibration curves were linear (r > 0.99) between 7.8 and 125 μg/ml.

C19-9 solution in DMSO was spiked in the plasma of mouse at 50 μg/ml concentration and kept in shaking water bath at 37 ◦C. Samples were collected at time points 0, 1, 2, 4, and 8 h, and the reaction was terminated with ice-cold acetonitrile containing internal standard. All the collected samples were stored below -20 ◦C until analysis. For analysis, the samples were thawed, vortexed, and centrifuged. The resulting supernatant was analyzed using LC-MS/MS method.

Incubations were conducted in 1× phosphate-buffered saline (1 × PBS, pH 7.4) containing 1ug C19-9 and 0.1mg mouse, rat, dog, or human microsomes in a final volume of 190 μl. After a 5 min pre-incubation at 37 °C, 10 μl of 20 mM NADPH was added (final concentration 1.0 mM), and incubation was continued for 30 min, 1h, 2h, 4h with gentle shaking. Incubations without NADPH were used as controls. Reactions were terminated by adding 200 μl of ice-cold acetonitrile and vortexing for 30 s and then centrifuged at rcf 15 000 for 15 min. Each supernatant was transferred to an autosampler vial, and 5.0 μl was injected onto LC-MS/MS system for analysis.

18. Statistics

All data are expressed as means ± standard deviations (S.D.) from at least three independent experiments. Statistical analyses were performed using the two-tailed Student's t-test to detect differences between the groups. P-values less than 0.05 were considered statistically significant.


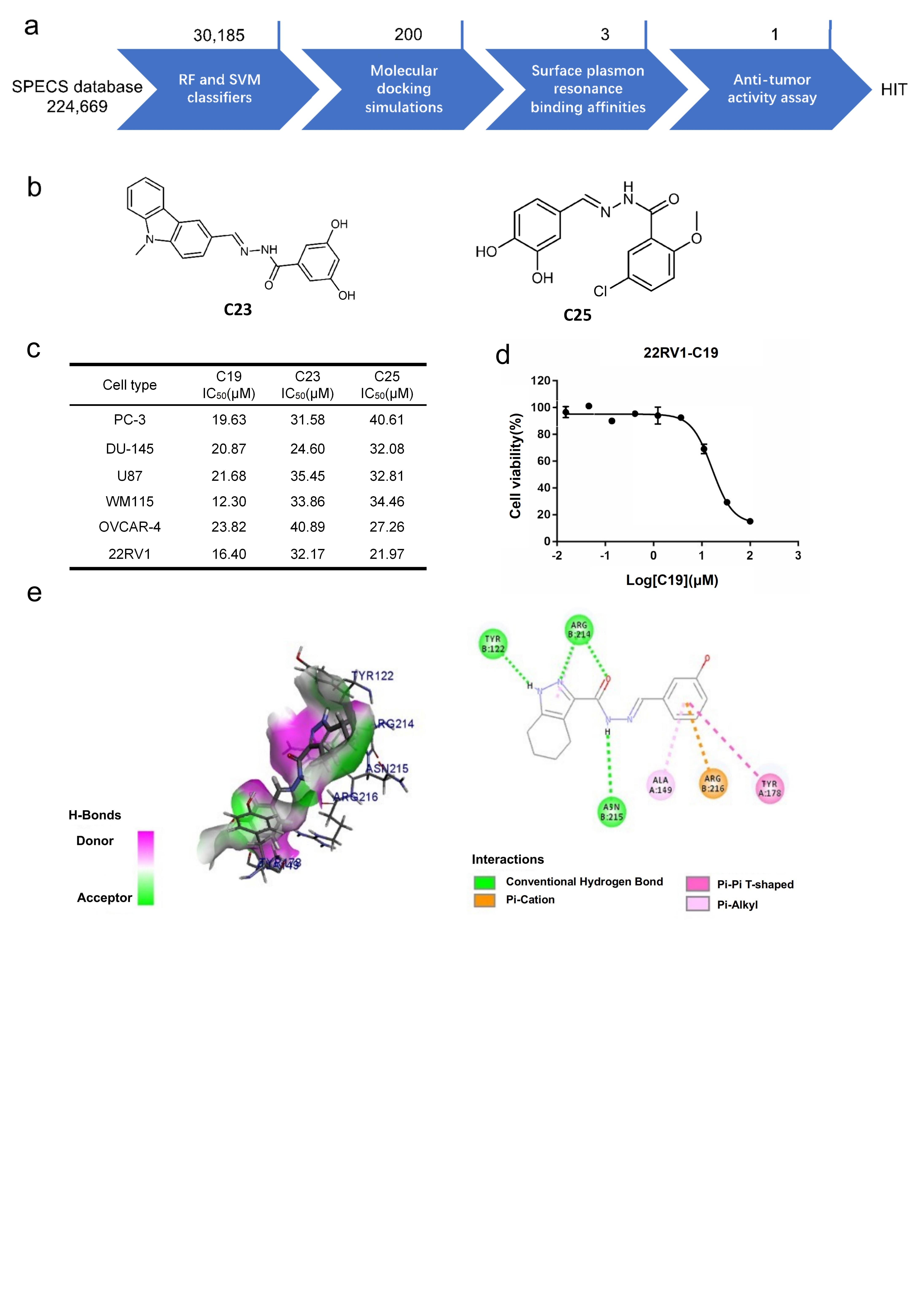


Figure.S1.

Identification of C19 as hit compound for αvβ3 inhibitor.  **a** The outline for the discovery of novel αvβ3 inhibitors. **b** The structures of C23 and C25. In SPR evaluation, C19, C23, and C25 had a good affinity with αvβ3 with a threshold of KD value less than 5μM. **c** The anti-tumor activity of C19, C23, and C25 on various cell lines in the Celltiter-Glo assay. **d** The Celltiter-Glo assay of evaluation of anti-tumor proliferation ability of C19 on 22RV1 at 24h. **e** Molecule docking of C19 with αvβ3.


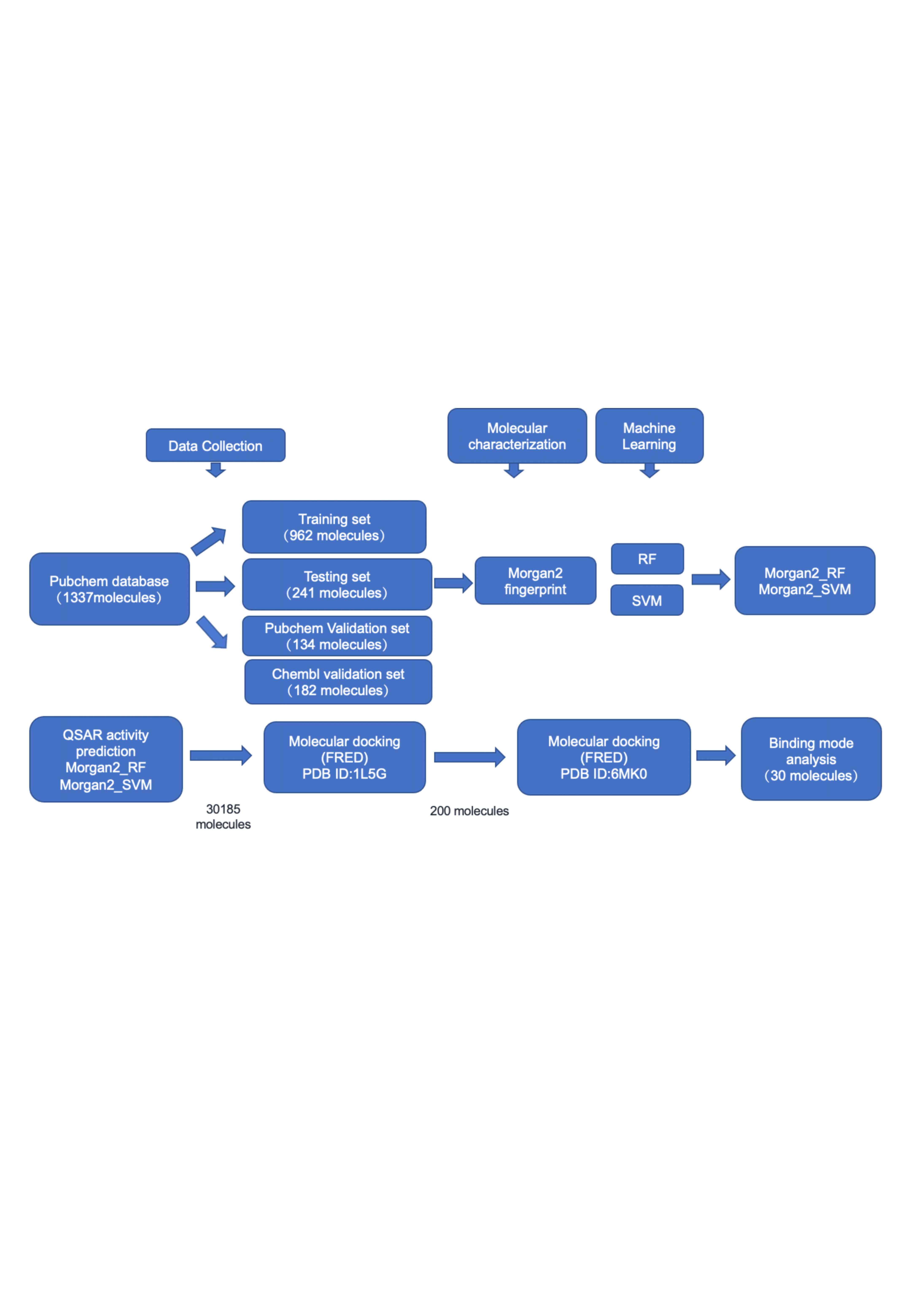


Figure.S2.

Identification of integrin αvβ3 inhibitor via structure-based VS (SBVS) and ligand-based VS (LBVS) combinatorial approaches of machine learning and molecular docking simulations with Specs in-house screening compound repository.

**
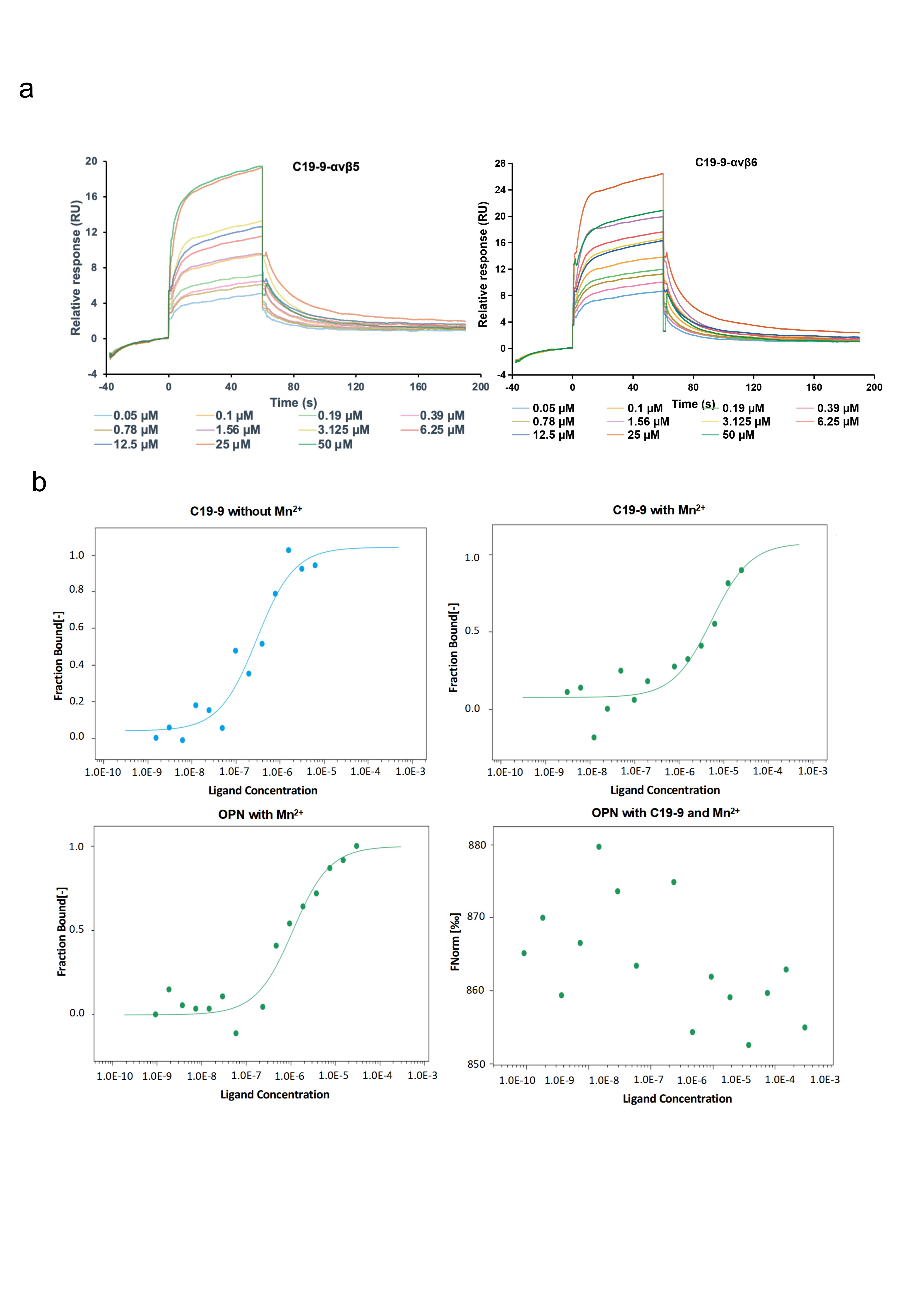
**

Figure.S3.

The affinity evaluation of C19-9. **a** The binding affinity of C19-9 with αvβ5 and αvβ6 tested SPR. **b** The detection of high-affinity interactions using MicroScale Thermophoresis. The change in thermophoresis is expressed as the change in the normalized fluorescence (DFnorm), which is defined as Fhot/Fcold. Titration of the non-fluorescent ligand results in a gradual change in thermophoresis, which is plotted as DFnorm to yield a binding curve, which can be fitted to derive binding constants. Top left: The interaction of C19-9 with αvβ3 was monitored in absence of Mn^2+^. The KD value was 0.287 μM. Top right: The interaction of C19-9 with αvβ3 was monitored in presence of Mn^2+^. The KD value was 6.37 μM. Bottom left: The interaction of OPN with αvβ3 was monitored in presence of Mn^2+^. Bottom right: The interaction of OPN with αvβ3 (after incubation with C19-9 for 2h) was monitored in presence of Mn^2+^.


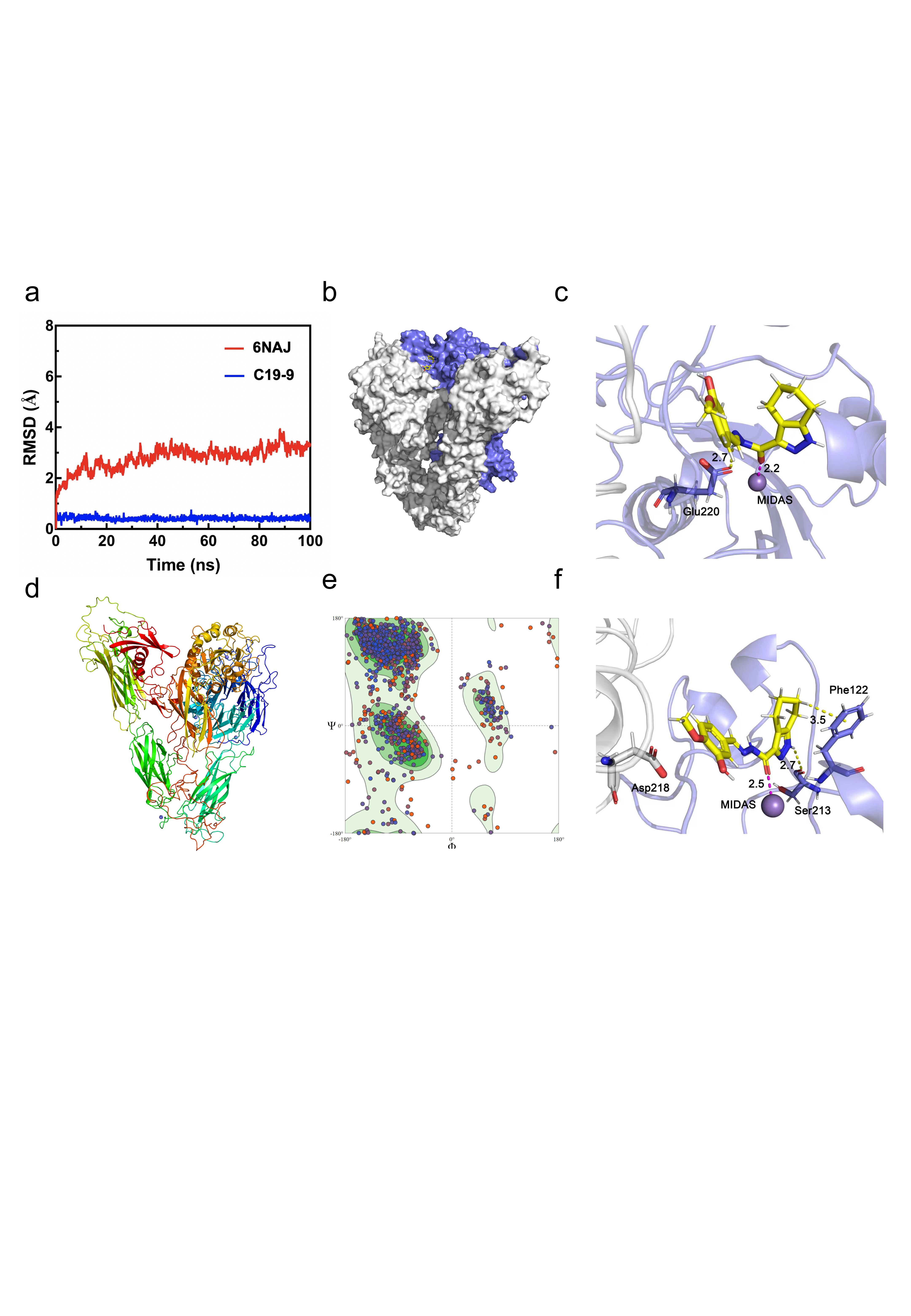


Figure.S4.

MD simulation of small molecule C19-9 with integrin avβ3.**a** System flexibility analysis of complex C19-9 with integrin avβ3. **b** The 3D binding mode of C19-9 with integrin avβ3 which was mutated D218A on av domain and mutated Y122A on β3 domain. **c** The 3D binding mode of mutated integrin avβ3 with C19-9. The backbone and residue of the mutated integrin avβ3 alpha chain are colored in white, and the backbone and residue of the mutated integrin avβ3 beta chain are colored in light blue. C19-9 is colored in yellow, and the Mn^2+^ in MIDAS site is shown as a purple sphere. The surrounding residues in the binding pockets are shown as stick. The yellow dashes represent hydrogen bond interaction, the magentas represent metal contact. **d** The homology model of mouse integrin avβ3. Models are computed by the SWISS-MODEL server homology modeling pipeline, which relies on ProMod3, a comparative modeling engine based on OpenStructure. **e** Ramachandran plot for mouse integrin avβ3. The mouse integrin avβ3 is colored by the spectrum of rainbow cartoon. Dark blue dots represent the residues in favored regions; orange dots represent the residues in allowed regions. **f** The 3D binding mode of C19-9 and mouse integrin avβ3.


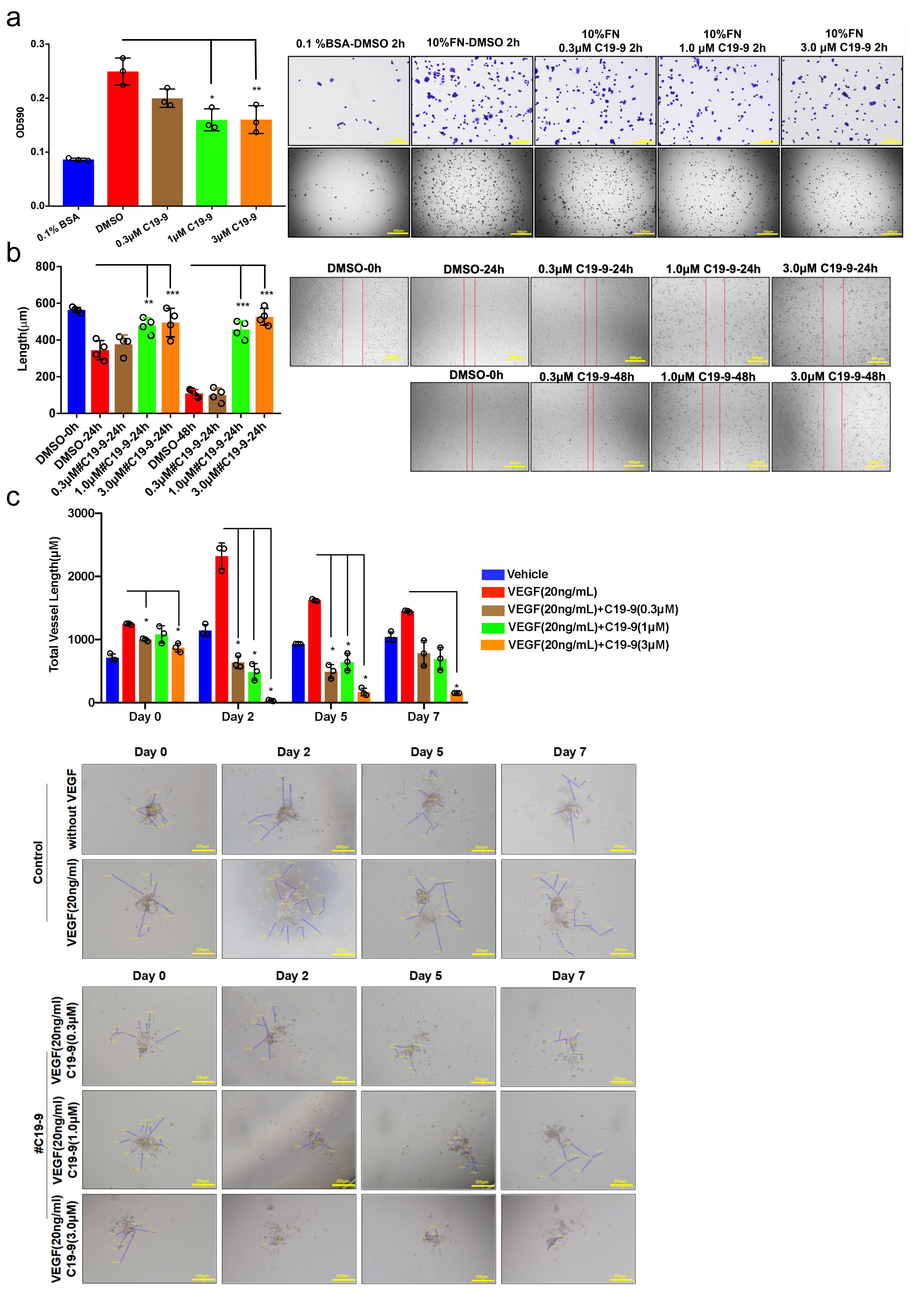


Figure.S5.

Evaluation of C19-9 on the inhibition of adhesion, migration and angiogenesis *in vitro*. **a,b** Inhibition of adhesion ability and migration ability of C19-9 on PC-3 cells with the concentration ranging from 0.3μM to 1μM. Adherent cells were fixed and stained with crystal violet as described in the supplement methods file. After solubilization, absorbance was measured at 590 nm. The cell movement into the wounded area was quantified by measuring the width of the cell-free zone at six distinct positions. Bars represent 200 μm and 500 μm. respectively. **c** Inhibition of HUVECs tube formation by C19-9 with the concentration ranging from 0.3μM to 1μM in the tumor organoid-induced angiogenesis assay. After 2 days, 5 days, and 7 days, tube formations were recorded with an inverted microscope and the tubular structures were counted manually. The diameter of organoids was measured using ImageJ. Scale bars, 200 µm. Data are expressed as mean ± SD, *P-value < 0.05, **P-value <0.01, ***P-value < 0.001.

**
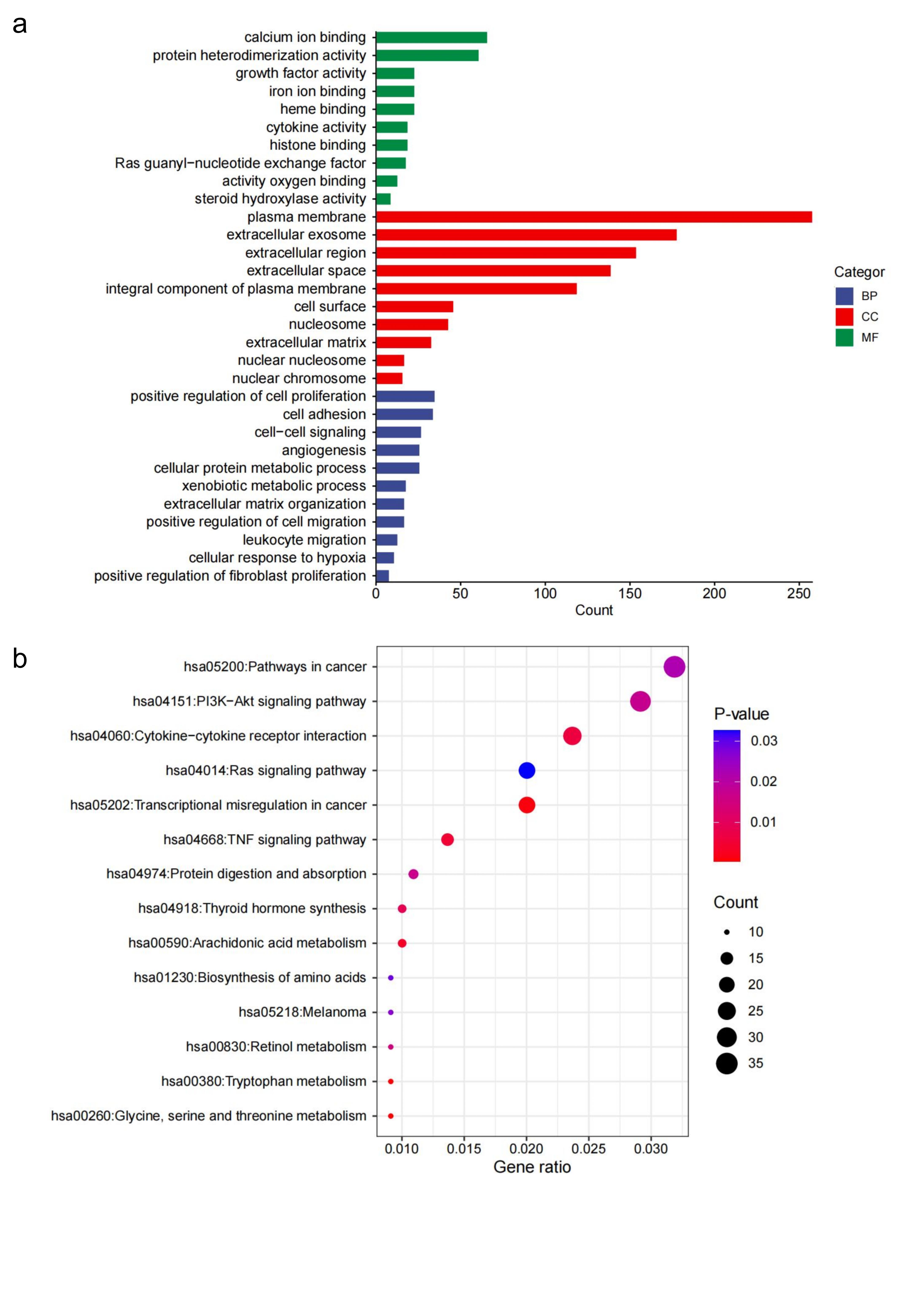
**

Figure.S6

Pathway enrichment analysis. GO(**a**)and KEGG (**b**) pathway analysis of differentially expressed genes after 22RV1 cells disposed of C19-9.


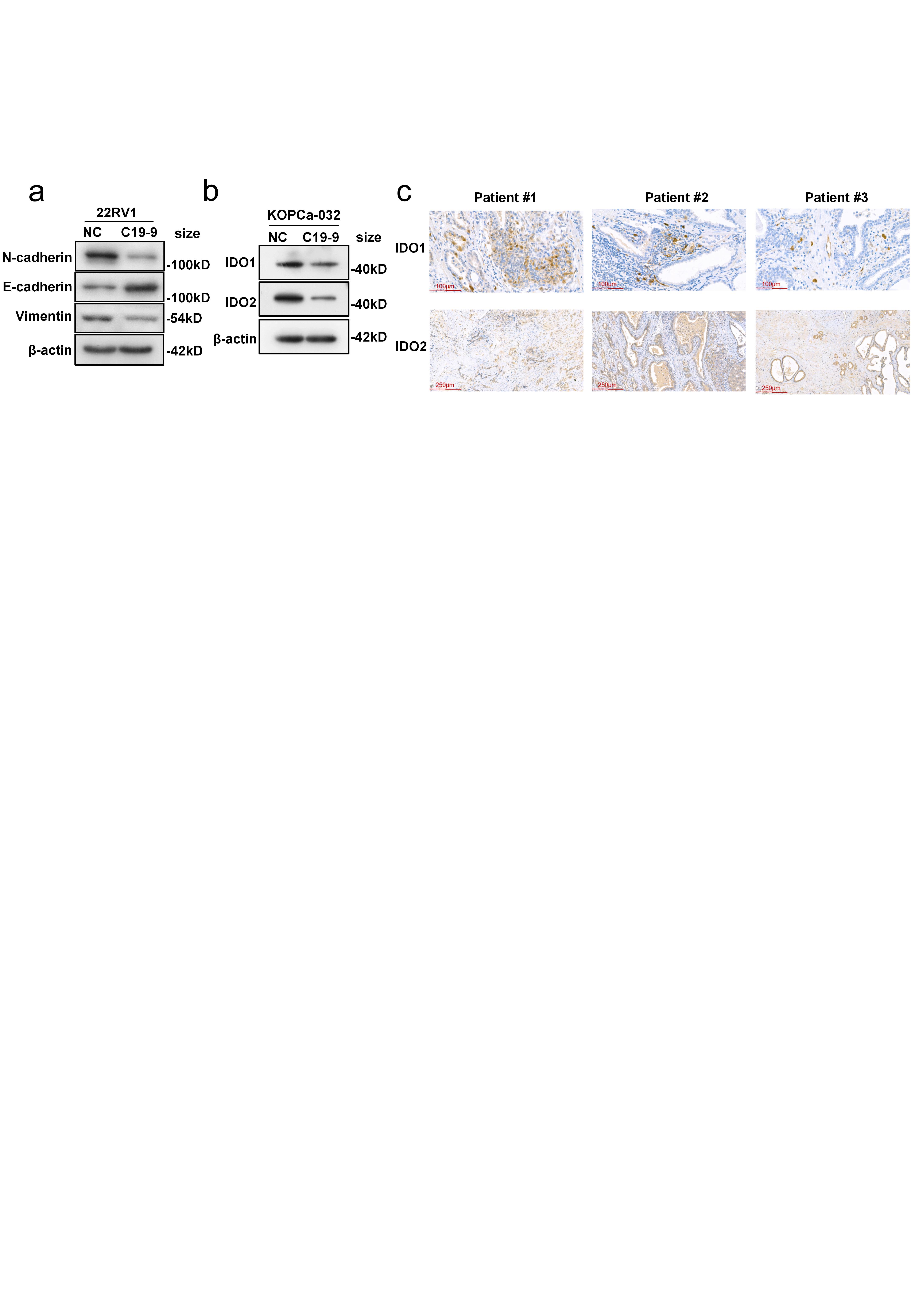
Figure.S7.

The effect of C19-9 on EMT and IDO expression. **a** EMT associated proteins (including E-cadherin, N-cadherin, and Vimentin) levels were detected by western blot. **b** The inhibition of IDO expression in PCa organoid KOPCa-032 after treatment with 0.5μM C19-9. **c** Histological analysis of IDO1 and IDO2 expression in PCa prostatectomy specimens (Scale bars, 100 µm and 250µm).


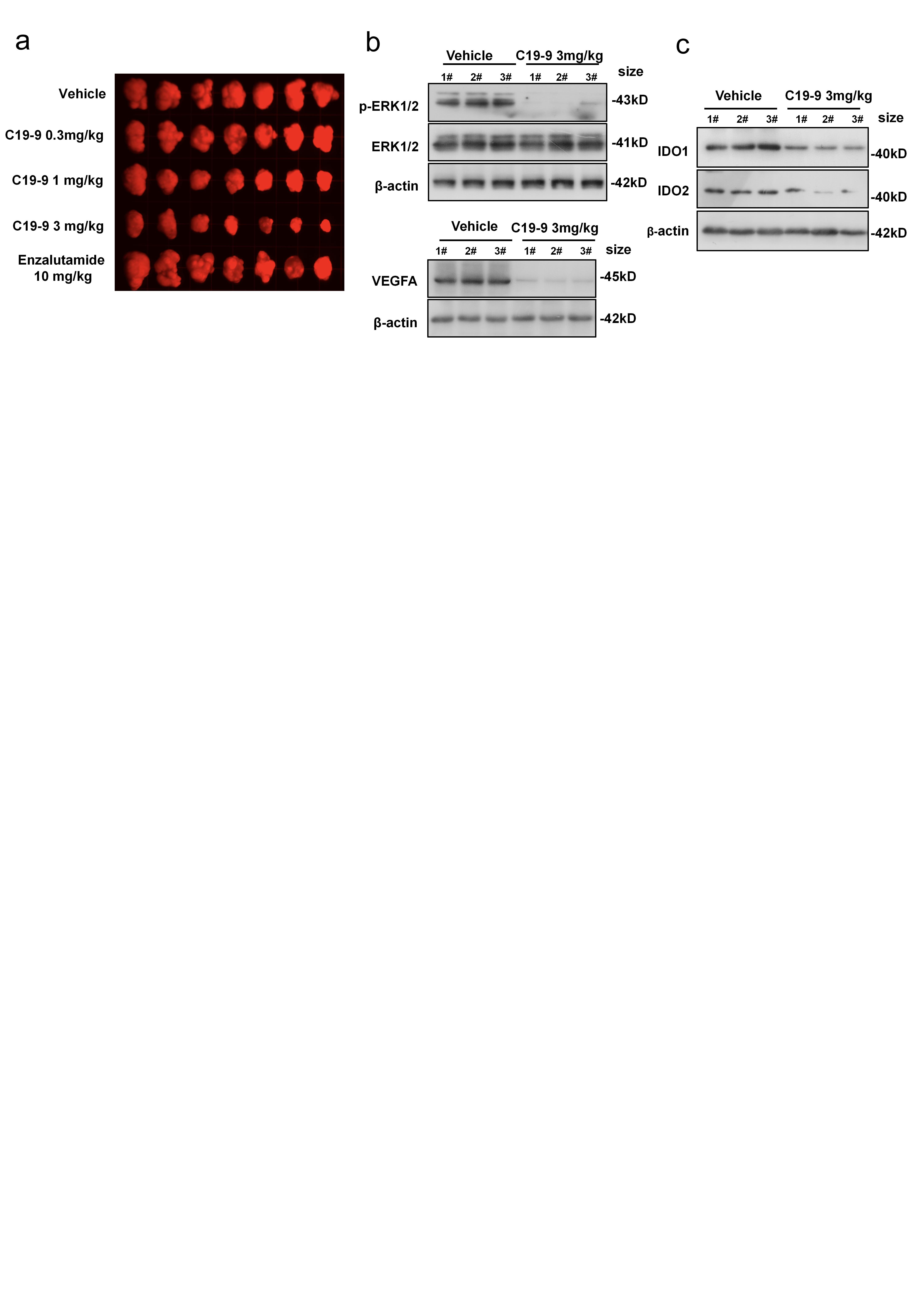


Figure.S8.

The efficacy and mechanism study of C19-9. **a** Image of tumors and in different groups after three weeks. **b,c** In the 22RV1-derived subcutaneous tumor growth xenograft model, tumor tissue was obtained after treatment for three weeks. IDO, VEGFA, and EKR1/2 signaling pathway related proteins were tested by western blot.


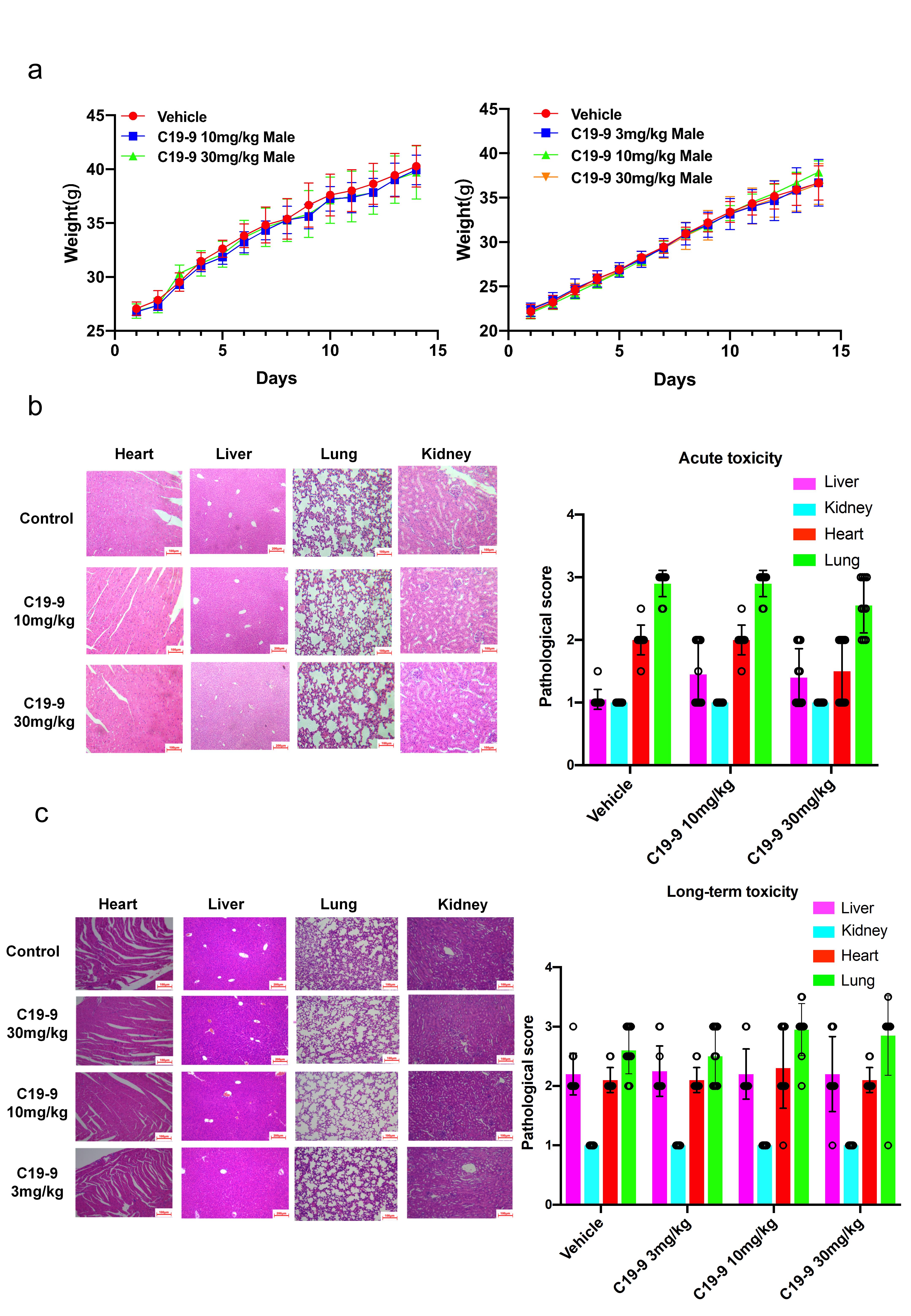


Figure.S9.

The toxicity studies of C19-9. **a** Toxicity studies of C19-9 after a single dose of 10mg/kg and 30mg/kg and with the dose of 3mg/kg, 10mg/kg, and 30mg/kg intraperitoneal administered continuously at 2 weeks. Body weight was monitored throughout the study. **b,c** The pathological score in review of sections of heart, liver, lungs, and kidney in long-term toxicity and acute toxicity assays. A necropsy was conducted on the day after the last administration. Pathological review of sections of heart, liver, lungs, and kidney(Scale bars, 100 µm and 200µm).


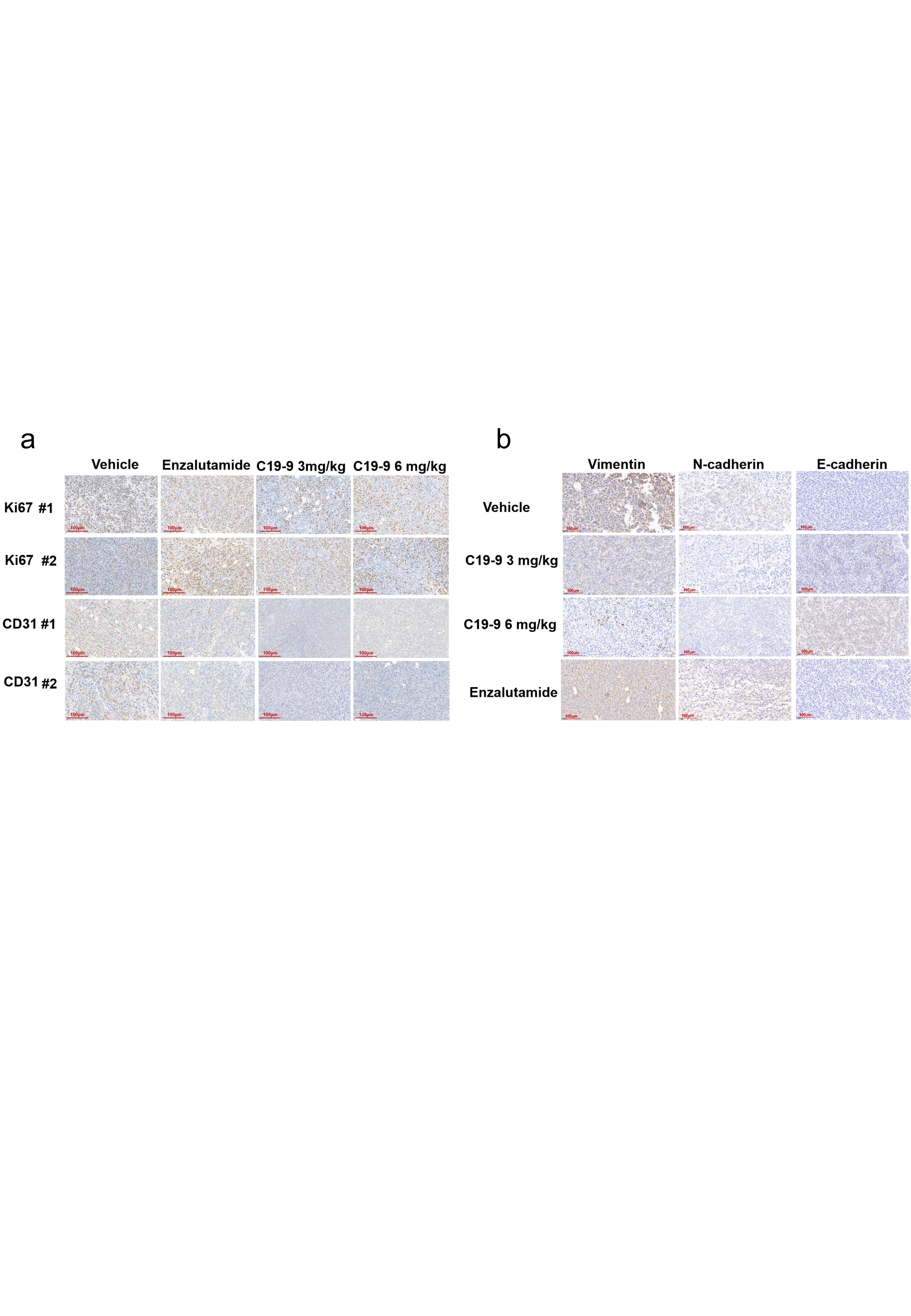


Figure.S10.

C19-9 inhibited CRPC progression *in vivo*. **a** Representative immunohistochemical images of Ki67 and CD31 in four groups (Scale bars, 100 µm). **b** Representative immunohistochemical images of Vimentin, N-cadherin, and E-cadherin in Vehicle, C19-9(3mg/kg, 6mg/kg), and Enzalutamide groups (Scale bars, 100 µm).

Figure.S11.

The various cytokine levels were determined by Luminex Assays (R&D Systems). Data are expressed as mean ± SD, *P-value < 0.05, **P-value <0.01, ***P-value < 0.001


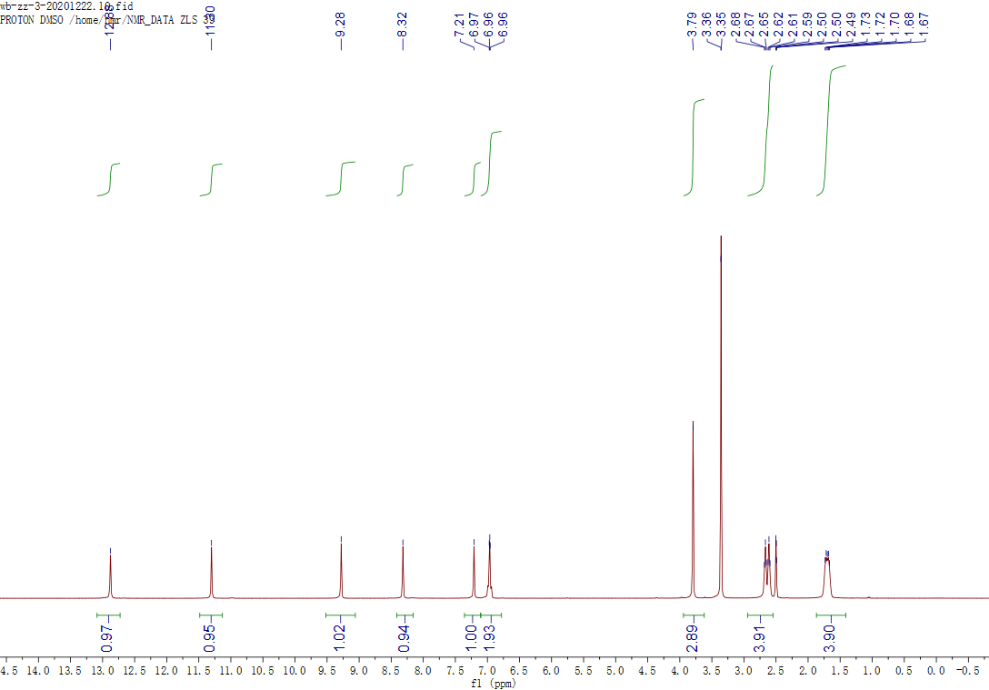

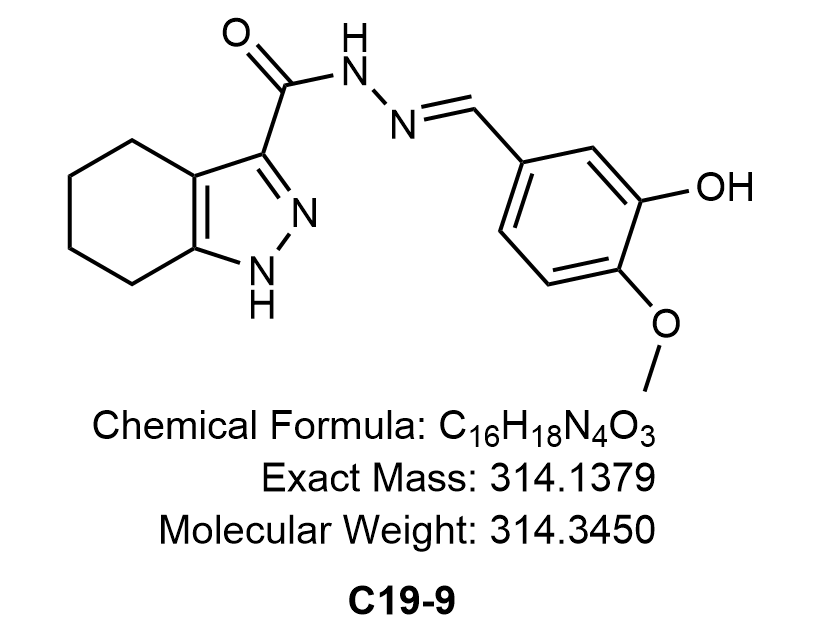


Figure.S12a.

HNMR of C19-9.


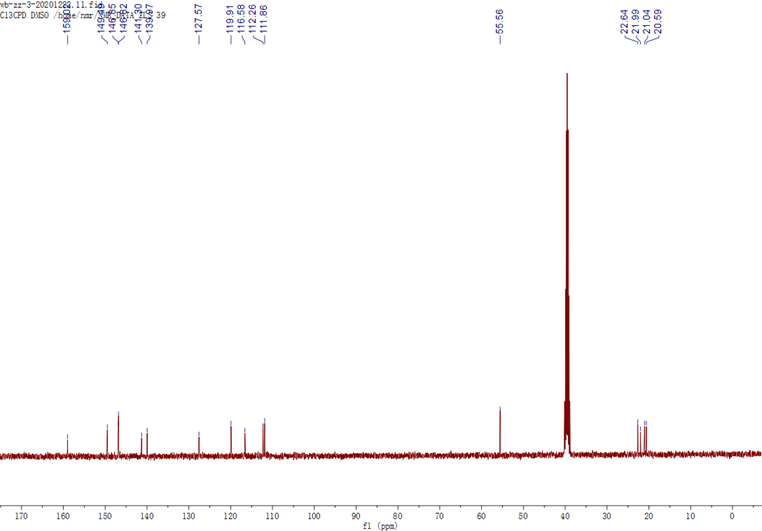

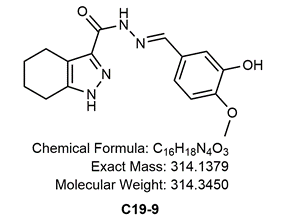


Figure.S12b.

CNMR of C19-9.

Figure.S12c.


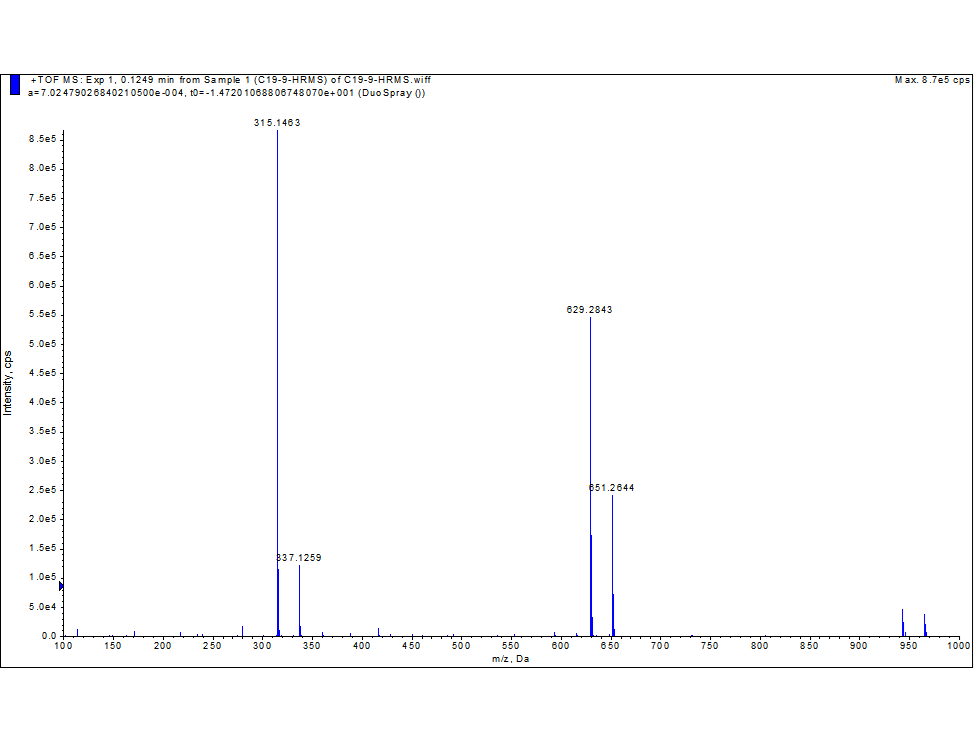

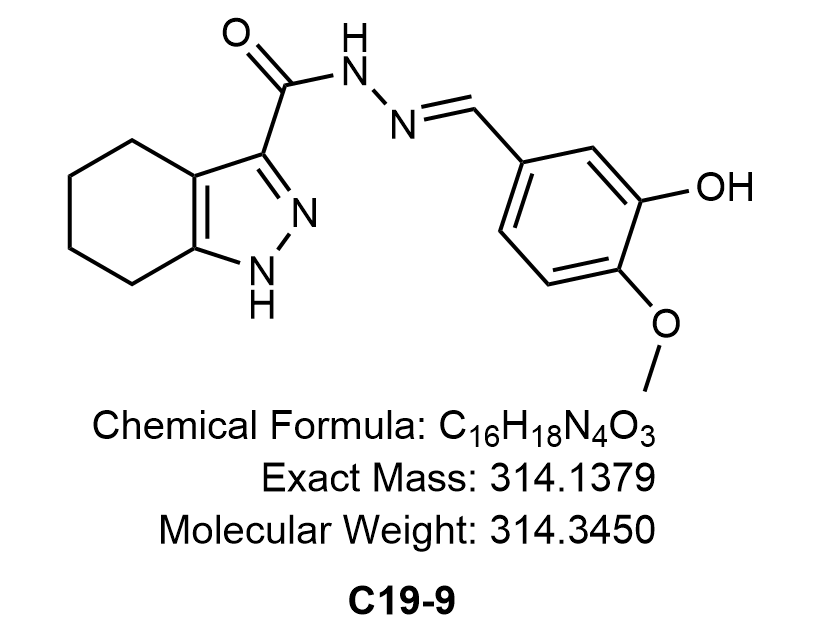


[M+H]^+^

HRMS of C19-9.


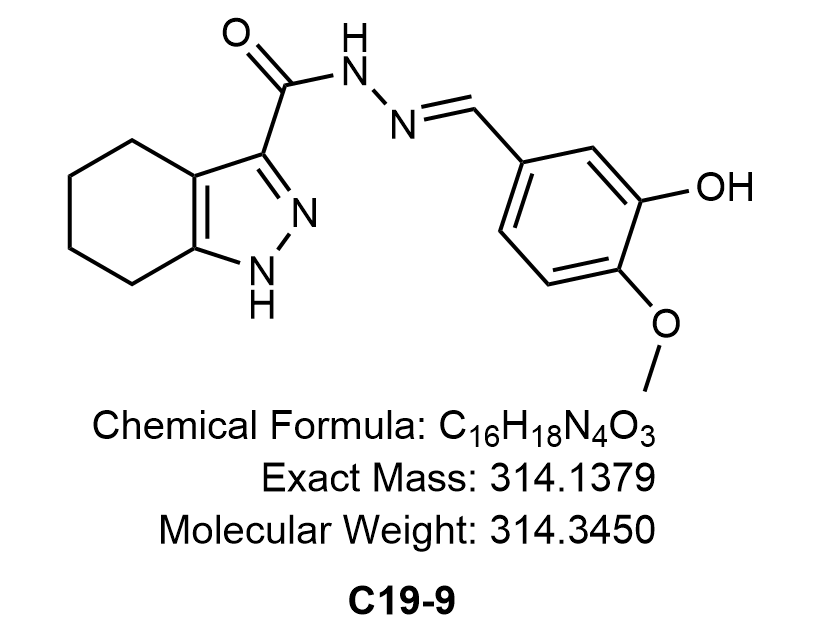

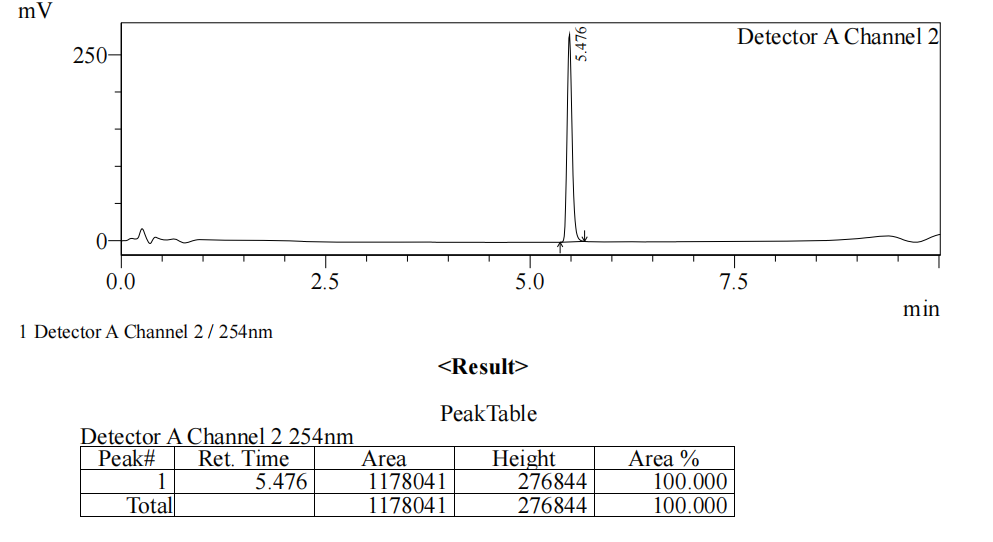


Figure.S12d.

HPLC spectrum of C19-9.

**Table S1.** The performance of random forest (RF) and support Vector Machine (SVM) classifiers based on Morgan2 fingerprints

Table S2. ∆Gtotal Obtained from the MM-GBSA Calculation for the C19-9-integrin avβ3 complex.
